# Clinical features, management, and outcomes of pulmonary mucormycosis: a decade-long retrospective study from a single center in central China

**DOI:** 10.3389/fmed.2026.1844463

**Published:** 2026-06-11

**Authors:** Chengqing Yang, Chunlin Mei, Tanze Cao, Minhui Mei, Shufang Chen, Xiuping Liu, Chao Quan, Xuan Wang

**Affiliations:** Wuhan Pulmonary Hospital (Wuhan Institute for Tuberculosis Control), Hubei Branch (Wuhan Pulmonary Hospital) of the National Clinical Research Center for Infectious Diseases, Wuhan, China

**Keywords:** diabetes mellitus, diagnostic strategies, imaging features, pathological diagnosis, pulmonary mucormycosis

## Abstract

**Background:**

Pulmonary mucormycosis is a life-threatening invasive fungal infection with challenging, often delayed diagnosis and high mortality. This study aims to elucidate the clinical characteristics, diagnostic and therapeutic approaches, and outcomes of pulmonary mucormycosis.

**Methods:**

This retrospective study enrolled inpatients diagnosed with pulmonary mucormycosis from January 2015 to May 2025. Clinical data were systematically collected and analyzed.

**Results:**

A total of 16 patients with pulmonary mucormycosis were included in the study. The mean age of the cohort was 52.50 ± 15.02 years, with a male predominance of 68.75%. Diabetes mellitus was identified as the most prevalent underlying condition, affecting 81.25% of patients. The most common clinical symptoms included cough (100%), expectoration (81.25%), fever (68.75%), chest tightness (68.75%), hemoptysis (50%), and chest pain (50%). Common imaging findings comprised nodules <3 cm (87.50%), consolidation (87.50%), ground-glass opacities (75.00%), halo sign (62.50%), and pleural effusion (50.00%). Of the 16 patients, 68.75% exhibited bronchial involvement. Twelve patients were confirmed by histopathology, and one by culture of sterile pleural fluid; the remaining three were classified as probable cases based on clinical and radiological findings, combined with detection of mucorales by mNGS or from non-sterile specimen cultures. The most frequently identified pathogen was Rhizopus microsporus. Systemic antifungal therapy was administered to 13 patients (81.25%), primarily involving a combination of amphotericin B formulations and azoles. Seventy-five percent of patients underwent bronchoscopic debridement or surgical intervention. The 12-month survival rate was 81.25%, with three patients succumbing to sepsis, massive hemoptysis, or respiratory failure, two of whom had extrapulmonary involvement. In this series, favorable outcomes were observed in patients who received combination therapy, particularly bronchoscopic debridement coupled with antifungal medication.

**Conclusion:**

Pulmonary mucormycosis is highly lethal, frequently associated with bronchial involvement. Bronchoscopy plays a crucial role in both diagnosis and treatment. Combined surgical/bronchoscopic intervention with systemic and local antifungal therapy may contribute to favorable outcomes in this series.

## Introduction

Mucormycosis is a rare, opportunistic, and life-threatening fungal infection caused by fungi belonging to the order Mucorales, including genera such as Rhizopus, Mucor, and Rhizomucor. This disease predominantly affects immunocompromised patients, particularly those with poorly controlled diabetes, hematologic malignancies, organ transplantation, trauma, burns, or those undergoing corticosteroid therapy (1–3). It presents with various clinical manifestations and may involve the rhino-orbital-cerebral region, lungs, skin, gastrointestinal tract, or even lead to disseminated infection (4). Pulmonary mucormycosis (PM) is a significant and common clinical type of mucormycosis, primarily occurring in immunocompromised populations. Common symptoms include fever, chest pain, dyspnea, and hemoptysis, which can be massive and fatal due to fungal invasion of blood vessels, leading to hemorrhage (5). This invasive infection may spread contiguously, affecting adjacent structures such as the bronchi, heart, and mediastinum. Studies indicate that the mortality rate of PM ranges from 48 to 87% (6–8), highlighting an extremely high fatality rate (9). Furthermore, the early symptoms of PM lack specificity and are easily confused with those of other pulmonary infections, which can lead to missed diagnoses, misdiagnoses, and delayed treatment.

Given the diagnostic challenges and poor prognosis associated with PM, we conducted this retrospective study to systematically summarize the demographic characteristics, clinical manifestations, laboratory findings, pathogenic results, treatment strategies, and clinical outcomes of 16 patients with PM, aiming to provide a clinical reference for the prognostic evaluation of this disease.

## Methods

### Study population

We conducted a retrospective analysis of patients diagnosed with PM at Wuhan Pulmonary Hospital between January 2015 and May 2025. Relevant medical records, including demographic data, medical history, clinical characteristics, laboratory findings, imaging features, treatment regimens, and clinical outcomes, were systematically collected and summarized for analysis. This study was approved by the Ethics Committee of Wuhan Pulmonary Hospital (Approval No.: 2025020). As a retrospective study, the requirement for written informed consent was waived.

### Inclusion criteria for pulmonary mucormycosis

All cases diagnosed with mucormycosis in the hospital were retrospectively identified. Subsequently, examination reports and medical records were systematically reviewed. The diagnostic criteria for included patients with PM encompassed both proven and probable cases. We adopted the classification criteria for proven, probable, and possible invasive fungal diseases from the European Organization for Research and Treatment of Cancer/Mycoses Study Group (EORTC/MSG), in combination with the Chinese Expert Consensus on Diagnosis and Management of Mucormycosis (2022) (10, 11). Proven cases were defined by histopathological confirmation or positive culture of Mucorales from sterile tissue specimens. Tissue specimens obtained via surgery or biopsy were subjected to special staining (e.g., hematoxylin and eosin, periodic acid–Schiff, or Grocott’s methenamine silver staining) and subsequently examined under a light microscope. The presence of characteristic mucoraceous hyphae—which are broad, aseptate or pauciseptate, and exhibit irregular, right-angle branching—was considered diagnostic for mucormycosis. Probable cases required the presence of host risk factors accompanied by clinical and imaging features suggestive of mucormycosis, plus at least one of the following: positive culture from non-sterile site specimens, or positive molecular biological testing, such as metagenomic next-generation sequencing (mNGS) or mucorales-specific PCR (12). For patients classified as probable cases, testing was conducted to exclude pulmonary tuberculosis through various methods, including acid-fast bacillus smear, mycobacterial culture, Xpert MTB/RIF, and T-SPOT. TB. Additionally, aspergillosis was ruled out using galactomannan testing and Aspergillus PCR on bronchoalveolar lavage fluid (BALF). Possible cases of pulmonary malignancy (PM) are characterized by the presence of host risk factors and compatible clinical or radiological features, albeit lacking any microbiological evidence.

### Diagnostic techniques

Histopathological examination was performed on formalin-fixed, paraffin-embedded tissue sections stained with hematoxylin and eosin (H&E), periodic acid–Schiff (PAS), and Grocott’s methenamine silver (GMS). Fungal culture was conducted on Sabouraud dextrose agar incubated at 28 °C. Metagenomic next-generation sequencing (mNGS) was performed on bronchoalveolar lavage fluid samples using the Illumina NextSeq platform.

### Outcome definitions

Outcome status was assessed at 12 months post-discharge jointly by the attending physicians and the study investigators, based on comprehensive clinical evaluation and chest CT findings. Cured was defined as complete resolution of all clinical symptoms, signs, and radiological abnormalities attributable to the infection, or the presence of only residual inactive lesions (e.g., fibrotic scars), with no evidence of relapse after discontinuation of antifungal therapy. Stable was defined as significant improvement in clinical symptoms and signs, with a reduction in radiological lesion size of ≥25% compared with baseline, without complete disappearance, and no emergence of new lesions.

### Statistical analysis

Data analysis was performed using SPSS version 23. Continuous variables are expressed as mean ± standard deviation (SD), while categorical variables are presented as frequencies and percentages. Non-normally distributed data are reported as median (interquartile range).

## Results

### Demographic and clinical characteristics of patients with pulmonary mucormycosis

This study included a total of 16 patients diagnosed with PM. The mean age of the cohort was 52.50 ± 15.02 years, with a predominance of male patients (68.75%). The most prevalent underlying condition was diabetes mellitus, which affected 81.25% of the patients, followed by concurrent pulmonary tuberculosis (18.75%) and renal insufficiency (12.5%). Hematologic malignancies were less frequently observed, with only one case each of acute lymphoblastic leukemia and acute myeloid leukemia (6.25% each). Granulocytopenia within 3 months prior to diagnosis was noted in 12.5% of the patients. Key laboratory findings indicated that the mean blood glucose level of the 16 patients was 11.64 ± 6.76 mmol/L, and among the 13 diabetic patients, the glycated hemoglobin (HbA1c) levels were elevated (10.8 ± 1.91%). Almost all patients (93.75%) presented with anemia (hemoglobin <130 g/L), and hypoalbuminemia (30.83 ± 6.02 g/L) was universally observed. Inflammatory markers were notably elevated, including C-reactive protein (CRP) (59.78 ± 53.27 mg/L) and erythrocyte sedimentation rate (ESR) (77.31 ± 38.15 mm/h). Neutrophilia (>6.3 × 10^9^ /L) and lymphopenia (<1 × 10^9^ /L) were each observed in 37.5% of the patients. Procalcitonin (PCT) levels were generally low in the majority (62.50%) of patients (see Table 1).

**Table 1:** Demographic and clinical characteristics of patients with pulmonary mucormycosis.

Characteristics	*N* = 16
Age, years	52.50 ± 15.02
Gender	
Male	11/16 (68.75%)
Female	5/16 (31.25%)
Height, m	1.66 ± 0.05
Weight, kg	59.31 ± 12.56
Time from symptom onset to diagnosis (days), median (IQR)	37.5 (14.0, 108.0)
Comorbidities, n/N (%)	
Acute lymphoblastic leukemia	1/16 (6.25%)
Acute myeloid leukemia	1/16 (6.25%)
Renal insufficiency	2/16 (12.50%)
Diabetes mellitus	13/16 (81.25%)
Sinusitis	2/16 (12.50%)
Solid organ tumor	1/16 (6.25%)
Hepatitis B	2/16 (12.50%)
Concurrent pulmonary tuberculosis	3/16 (18.75%)
Granulocytopenia within 3 months before diagnosis	2/16 (12.50%)
Surgery within past 3 months	2/16 (12.50%)
Glucocorticoid treatment within 3 months before diagnosis	2/16 (12.50%)
Laboratory parameters	
White blood cell count (>9.5 × 10^9^/L)	4/16 (25.00%)
Neutrophil count (> 6.3 × 10^9^/L)	6/16 (37.50%)
Lymphocyte count (<1 × 10^9^/L)	6/16 (37.50%)
Hemoglobin (<130 g/L)	15/16 (93.75%)
Platelet count (>350 × 10^9^/L)	2/16 (12.50%)
HbA1c, % (>6.0%)	13/13 (100%)
HbA1c, % (*n* = 13)	10.8 ± 1.91
ALB (<40 g/L)	16/16 (100%)
ALB (g/L)	30.83 ± 6.02
Blood glucose (> 6.1 mmol/L)	11/16 (68.75%)
Blood glucose (mmol/L)	11.64 ± 6.76
LDH (>250 U/L)	4/16 (25.00%)
C-reactive protein (>5 mg/L)	59.78 ± 53.27
ESR (>20 mm/h)	77.31 ± 38.15
PCT (<0.15 ng/mL)	10/16 (62.50%)

HbA1c, Glycated Hemoglobin; ALB, Albumin; LDH, Lactate Dehydrogenase; CRP, C-reactive protein; ESR, Erythrocyte Sedimentation Rate. Results in the table are calculated with *N* = 16 unless otherwise indicated; continuous variables: mean ± SD; categorical variables: frequencies and percentages; non-normally distributed data: median (IQR).

All 16 patients (100%) exhibited cough, making it the most common symptom, followed by sputum production (81.25%), fever, and chest tightness (68.75% each). Hemoptysis and chest pain were each observed in 50% of the patients. Dyspnea was present in 43.75% of cases, while cyanosis and pulmonary crackles were less common (6.25%). Notably, other symptoms, such as abdominal pain and rash, were reported by the vast majority of patients (93.75%) (Table 2). Regarding the sites of involvement, the lung was universally affected (100%), with frequent bronchial involvement (68.75%). Pleural involvement was noted in 25% of patients, and extrapulmonary dissemination to sites including the pleural cavity (18.75%), small intestine (12.50%), colon (6.25%), and abdominal cavity (6.25%) was also observed (Table 2).

**Table 2:** Clinical manifestations of pulmonary mucormycosis.

Variable	All patients (*n* = 16)
Symptoms	
Fever	11 (68.75%)
Chest tightness	11 (68.75%)
Chills	10 (62.50%)
Cough	16 (100%)
Sputum production	13 (81.25%)
Hemoptysis	8 (50%)
Chest pain	8 (50%)
Dyspnea	7 (43.75%)
Cyanosis	1 (6.25%)
Pulmonary crackles	1 (6.25%)
Other symptoms (e.g., abdominal pain, rash)	15 (93.75%)
Involved sites	
Lung	16 (100%)
Bronchus	11 (68.75%)
Pleural cavity	3 (18.75%)
Small intestine	2 (12.50%)
Colon	1 (6.25%)
Abdominal cavity	1 (6.25%)
Pleura	4 (25.00%)

“Other symptoms” included all atypical respiratory and systemic symptoms.

### Radiographic features of pulmonary mucormycosis

Radiographic evaluation revealed extensive pulmonary involvement characterized by a diverse array of features (Table 3). The most prevalent parenchymal abnormalities included nodules measuring less than 3 cm (87.50%), consolidation or patchy infiltration (87.50%), and ground-glass opacity (75%). Notably, multiple small nodules were common, with 64.29% (9/14) of patients exhibiting 10 or more nodules. Characteristic signs such as the halo sign (62.50%) and air bronchogram (56.25%) were frequently observed, while the reverse halo sign was less common (18.75%). Cavitation with intracavitary contents was identified in 31.25% of patients. Lobar involvement was predominantly multifocal: 31.25% of cases involved only a single lobe, while the remaining 68.75% had involvement of more than one lobe; 31.25% of cases exhibited involvement of all five lobes, and bilateral lung involvement was present in 43.75% of patients (Table 3).

**Table 3:** Radiographic features of pulmonary mucormycosis.

Radiographic feature	All patients (*n* = 16)
Lobe of lesion distribution	
Single lobe	5 (31.25%)
Two lobes	4 (25.00%)
Three lobes	2 (12.50%)
Five lobes	5 (31.25%)
Bilateral involvement	7 (43.75%)
Left upper lobe	8 (50.00%)
Left lower lobe	10 (62.50%)
Right upper lobe	9 (56.25%)
Right middle lobe	7 (43.75%)
Right lower lobe	10 (62.50%)
Imaging features	
Ground-glass opacity	12 (75%)
Nodule (<3 cm)	14 (87.50%)
Number of nodules (≥10)	9/14 (64.29%)
Nodule (≥3 cm)	6 (37.5%)
Consolidation/patchy infiltration	14 (87.50%)
Wedge-shaped infarction	2 (12.50%)
Atelectasis	7 (43.75%)
Subpleural distribution	3 (18.75%)
Peribronchovascular distribution	2 (12.50%)
Interlobular septal thickening	5 (31.25%)
Halo sign	10 (62.50%)
Reverse halo sign	3 (18.75%)
Cavitation	5 (31.25%)
Intracavitary fungus ball/hyphae	5 (31.25%)
Air bronchogram	9 (56.25%)
Tree-in-bud sign	1 (6.25%)
Lobulation	1 (6.25%)
Pleural indentation	6 (37.50%)
Pleural thickening	13 (81.25%)
Pleural effusion	8 (50.00%)
Mediastinal lymphadenopathy	4 (25.00%)
Pneumothorax	1 (6.25%)

**Figure 1:**
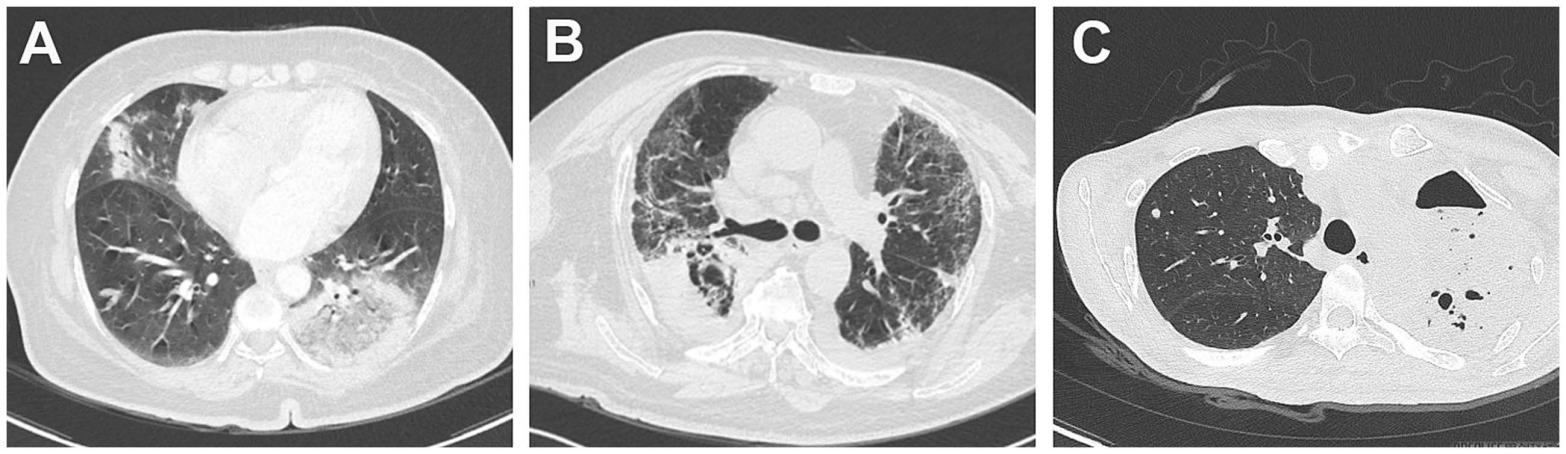
Characteristic chest CT imaging findings of pulmonary mucormycosis. **(A)** The reverse halo sign is observed in the left lower lobe, alongside multiple nodules exhibiting halo signs in the right middle lobe. **(B)** A cavity with septations in the right lower lobe, which evolved from a reverse halo sign, is accompanied by bilateral pleural effusions. **(C)** Small nodules are noted in the right upper lobe, with extensive consolidation and cavity formation in the left lung.

Pleural and less common features were also prominent (Table 3). Pleural thickening was the most frequently observed finding (81.25%), followed by pleural effusion (50.00%) and pleural indentation (37.50%). Other significant features included atelectasis (43.75%), interlobular septal thickening (31.25%), and mediastinal lymphadenopathy (25.00%). Features such as wedge-shaped infarction (12.50%), peribronchovascular distribution (12.50%), and the tree-in-bud sign (6.25%) were infrequently identified, reflecting the variable radiographic presentation of PM (Table 3). Figure 1 illustrates the typical radiological findings of PM.

### Diagnostic approaches of pulmonary mucormycosis

**Figure 2:**
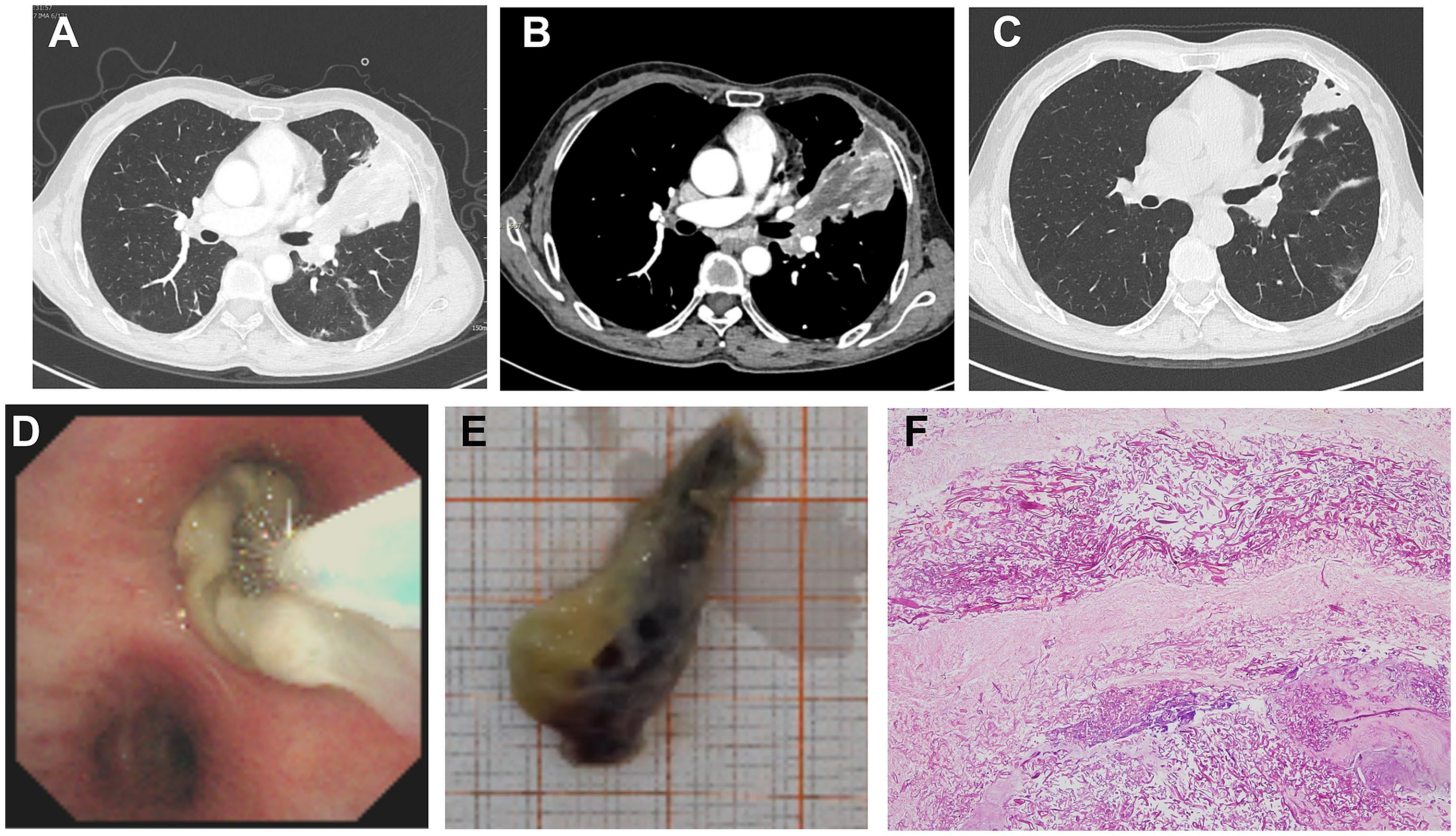
A case of pulmonary mucormycosis with characteristic imaging and bronchoscopic findings. **(A)** Chest CT performed on March 28, 2024, reveals a mass-like shadow in the left upper lobe. **(B)** Contrast-enhanced CT demonstrates a linear low-density mucus plug along the bronchial pathway within the mass-like shadow of the left upper lobe. **(C)** A follow-up chest CT taken on November 12, 2024, indicates significant resolution of the left upper lung lesion. **(D)** Bronchoscopy uncovers a necrotic mucus plug that completely obstructs the left upper bronchus. **(E)** Necrotic material is extracted during bronchoscopy. **(F)** Histopathological examination (H&E stain, ×100) of the necrotic material reveals fungal infection accompanied by suppurative inflammation, with the fungal morphology consistent with Mucor.

Among the 16 patients, 13 were confirmed cases and 3 were classified as probable cases. Of the 13 confirmed cases, 12 were definitively diagnosed with mucormycosis through histopathological examination. Pathological tissue specimens were obtained via bronchoscopy in 10 patients, while one specimen each was acquired through thoracoscopy and surgical resection. A representative case illustrating the bronchoscopic and imaging findings is presented in Figure 2. One confirmed case was diagnosed through the isolation and culture of Mucorales from sterile pleural effusion. The remaining three cases were deemed probable based on clinical manifestations, imaging findings, and the detection of Mucorales through mNGS or culture from non-sterile specimens. These three patients underwent systematic screening to exclude pulmonary tuberculosis (with Xpert, culture, and T-SPOT. TB all yielding negative results) and aspergillosis (with the BALF galactomannan test and Aspergillus PCR both negative). mNGS was performed on 6 patients (5 from bronchoalveolar lavage fluid and 1 from a tissue specimen), yielding positive results in all cases (100.0%). Among the 8 cases with identified species (by mNGS or culture), 6 were identified as Rhizopus microsporus, 1 as Rhizopus delemar, and 1 as Mucor racemosus (see Tables 4, 5).

**Table 4:** Diagnostic approaches of pulmonary mucormycosis.

Diagnostic approaches	All patients (*n* = 16)
Pathological biopsy	12
Bronchoscopy	10
Thoracoscopy	1
Surgical resection	1
Culture	3
Sterile sample culture	2
Sputum or BALF culture	1
mNGS detection	6
mNGS positive	6
Classification of mucor species	8
Rhizopus microsporus	6
Rhizopus delemar	1
Mucor racemosus	1

BALF, Bronchoalveolar Lavage Fluid; mNGS, Metagenomic Next-Generation Sequencing.

**Table 5:** Treatment and outcome of pulmonary mucormycosis.

ID	Age/sex	Underlying diseases	Complications	Diagnosis method	Diagnosis	Sites involved	mNGS	mNGS positive	Mucorale species	Hospital stay (days)	Treatment strategy	Systemic antifungal therapy	Outcome (12 months post-discharge)
1	48/M	DM, hepatitis B	Empyema	Pathology	Proven	Thorax, lung	No	NA	NA	66	Surgery + local AmB + systemic antifungals	ABCD (45d), Isavuconazole (6 m)	Cured
2	15/M	ALL	Pneumothorax, neutropenia	Pathology, BALF mNGS	Proven	Lung, bronchus	Yes	Yes	*Rhizopus microsporus*	102	Bronchoscopic clearance + local AmB + antifungals+Surgery	L-AmB (60d) + posaconazole (18 m)	Cured
3	63/M	CKD on dialysis	Intestinal obstruction, perforation	Pathology	Proven	Bowel, colon, abdomen, lung	No	NA	NA	20	Surgery + local L-AmB + antifungals	L-AmB (2d) + posaconazole (2d)	Death (sepsis)
4	71/M	DM, COVID-19	Respiratory failure, empyema	Clinical, imaging, culture (pleural fluid)	Proven	Lung, thorax, abdomen	No	NA	*Rhizopus microsporus*	25	Antifungals	Voriconazole (20 d) + caspofungin (14 d)	Death (respiratory failure)
5	45/M	DM	DKA	Pathology	Proven	Bronchus, lung	No	NA	NA	3	Bronchoscopic clearance	None	Death (massive hemoptysis)
6	47/M	DM	CKD	Clinical, imaging, BALF mNGS	Probable	Bronchus, lung, pleura	Yes	Yes	*Rhizopus microsporus*	5	Antifungals + drainage	Posaconazole (2 m)	Stable
7	52/F	DM	None	Pathology	Proven	Bronchus, lung, pleura	No	NA	NA	70	Bronchoscopic clearance + local AmB + antifungals	ABCD (60 d), Isavuconazole (12 m)	Cured
8	51/F	DM	None	Clinical, imaging, BALF mNGS	Probable	Lung	Yes	Yes	*Rhizopus microsporus*	7	Antifungals	L-AmB (7 d), Isavuconazole (2 m)	Stable
9	61/M	DM	None	Pathology, tissue culture	Proven	Bronchus, lung	No	NA	*Rhizopus microsporus*	14	Bronchoscopic clearance+antifungals	Posaconazole (6 m)	Cured
10	51/M	DM	None	Pathology	Proven	Bronchus, lung	No	NA	NA	14	Bronchoscopic clearance+antifungals	Posaconazole (2 m)	Cured
11	69/M	DM, post-lung cancer surgery, sinusitis	None	Pathology	Proven	Bronchus, lung, pleura	No	NA	NA	12	Bronchoscopic clearance+antifungals	Isavuconazole (6 m)	Stable
12	67/M	DM, TB	None	Pathology, BALF mNGS	Proven	Bronchus, lung, pleura	Yes	Yes	*Rhizopus delemar*	12	Bronchoscopic clearance+antifungals	Isavuconazole (3 m)	Stable
13	65/M	AML, hepatitis B, TB	Neutropenia	Pathology	Proven	Bronchus, lung	No	NA	NA	26	Bronchoscopic clearance+antifungals	Isavuconazole (3 m)	Stable
14	27/M	DM, TB	None	Pathology	Proven	Bronchus, lung	No	NA	NA	12	Bronchoscopic clearance+antifungals	Isavuconazole (3 m)	Stable
15	59/F	Bronchiectasis	None	Clinical, imaging, BALF mNGS, culture (sputum)	Probable	Bronchus, lung	Yes	Yes	*Rhizopus microsporus*	8	Antifungals	ABCD (7 d), Isavuconazole (3 m)	Stable
16	49/F	DM	DKA, PE, respiratory failure	Pathology, tissue mNGS	Proven	Bronchus, lung	Yes	Yes	*Mucor racemosus*	64	Bronchoscopic clearance + local AmB + antifungals	ABCD (50 d), Isavuconazole (12 m)	Cured

DM, Diabetes Mellitus; ALL, Acute Lymphoblastic Leukemia; AML, Acute Myeloid Leukemia; HD, Hemodialysis; TB, Tuberculosis; DKA, Diabetic Ketoacidosis; CKD, Chronic Kidney Disease; BALF, Bronchoalveolar Lavage Fluid; mNGS, Metagenomic Next-Generation Sequencing; AmB, Amphotericin B; L-AmB, Liposomal Amphotericin B; ABCD, Amphotericin B Colloidal Dispersion; PE, Pulmonary Embolism.

### Treatment and prognosis of pulmonary mucormycosis

Of the 16 patients, 13 (81.25%) received systemic antifungal therapy. The primary intravenous agents administered were liposomal amphotericin B (L-AmB) and amphotericin B colloidal dispersion (ABCD), each at a systemic dosage of 3–5 mg/kg/day. Oral medications included isavuconazole and posaconazole. The standard treatment regimen consisted of initial intravenous administration followed by sequential oral azole therapy. Among the 13 surviving patients, the median duration of systemic antifungal therapy was 90 days (range: 67 to 225 days). Twelve patients (75%) underwent interventional treatment, which included bronchoscopic debridement in 11 patients and surgical resection in 3 patients. Nine patients received bronchoscopic intervention within 48–72 h after the initiation of antifungal therapy. Additionally, five patients received local instillation of amphotericin B during the procedure, utilizing 10 mg of amphotericin B for injection dissolved in 5 mL of sterile water, administered either bronchoscopically or intrapleurally.

The overall 12-month post-discharge survival rate was 81.25% (13/16). Among the 13 survivors, 6 achieved clinical cure, while 7 remained in stable condition. Three patients (18.8%) died, primarily due to severe complications such as sepsis, respiratory failure, and massive hemoptysis (Table 5).

## Discussion

In this retrospective analysis of 16 patients with pulmonary mucormycosis (PM) from a specialized pulmonary hospital in central China over the past decade, diabetes mellitus emerged as the predominant predisposing condition (81.25%), consistent with patterns observed in developing countries, in contrast to developed nations where hematologic malignancies predominate (13–15). The most common clinical symptoms included cough, sputum production, fever, and chest tightness; hemoptysis occurred in 50% of patients, reflecting the angioinvasive nature of mucormycosis. However, these presenting symptoms remain nonspecific, complicating early diagnosis (16). Imaging studies revealed that nodules and consolidation were the predominant findings, with most patients presenting multiple small nodules. The halo sign was observed more frequently than the classically described reverse halo sign, and pleural involvement was common. Signs of airway dissemination (e.g., tree-in-bud) and tumor-like features (e.g., lobulation) were rare, which aids in differentiating mucormycosis from tuberculosis or lung cancer. Histopathologically, the halo sign reflects a central infarcted nodule surrounded by a rim of hemorrhage caused by Mucorales hyphae invading and thrombosing pulmonary vessels, directly demonstrating the angioinvasive nature of the infection (17). Multiple small nodules frequently correspond to multifocal hemorrhagic infarcts, and the reverse halo sign has been shown to consist of a central zone of coagulative necrosis with preserved alveolar air spaces, surrounded by an outer ring of organization and consolidation (18). This correlation between imaging and pathology not only explains the high incidence of hemoptysis but also underscores the diagnostic value of obtaining tissue from infarcted or necrotic areas to identify characteristic hyphae. Given the overlap of these imaging features with aspergillosis, other fungal infections, malignancies, and tuberculosis, early etiological confirmation is essential (19–23). All 13 diabetic patients in our cohort exhibited markedly elevated HbA1c levels, indicating poor long-term glycemic control. Notably, two of the three deceased patients presented with diabetic ketoacidosis (DKA). Prior studies have demonstrated a positive correlation between HbA1c levels and the severity of mucormycosis, with hyperglycemia and ketoacidosis facilitating fungal invasion through the upregulation of the endothelial GRP78 receptor and impairment of phagocyte function (24). Therefore, optimizing glycemic control and promptly correcting DKA are essential in managing pulmonary mucormycosis in diabetic patients.

Diagnosing mucormycosis remains a complex challenge. Laboratory methods utilized in clinical practice include histopathology, direct microscopy, and culture (25). Molecular testing can enhance detection and identification, serving as a valuable complement to traditional diagnostic approaches. In this study, the majority of patients were diagnosed through histopathology, with early histopathological examination facilitating prompt disease detection. In recent years, molecular techniques, particularly polymerase chain reaction (PCR) and mNGS (26), have gained increasing importance in the diagnosis of mucormycosis (27, 28). Notably, mNGS, when applied to bronchoalveolar lavage fluid or other clinical samples, demonstrated high sensitivity, with all six tested cases yielding positive results. As a culture-independent method capable of rapid identification of multiple pathogens, mNGS serves as a powerful and efficient diagnostic tool for critically ill patients with pulmonary mucormycosis presenting atypically, with negative conventional microbiological results, or contraindications to invasive procedures (29–31). Furthermore, given our observation that PM is frequently diagnosed with delays, we propose that mNGS may facilitate earlier and more accurate diagnosis in individuals with risk factors or clinical suspicion of mucormycosis. However, the potential for false-positive results due to contamination or detection of colonizing organisms should be carefully considered, and its relatively high cost currently limits routine clinical application. Therefore, mNGS is most appropriately used in patients with high clinical suspicion but negative conventional workup, or when timely tissue diagnosis is not feasible. Additionally, for patients in whom differentiation between Aspergillus and Mucorales infections is challenging, or for high-risk patients exhibiting bilateral lung involvement and poor responses to antibacterial therapy, empirical antifungal treatment should encompass coverage for mucormycosis. The most commonly identified pathogen in this study was Rhizopus microsporus, aligning with previous reports that designate Rhizopus species as predominant (32). However, variations in species distribution may be influenced by geographic location, host factors, and diagnostic sensitivity.

In this study, 81.25% of patients received systemic antifungal therapy, primarily utilizing amphotericin B formulations, including L-AmB and ABCD, in conjunction with azoles such as isavuconazole and posaconazole. Additionally, 75% of patients underwent interventional procedures, including bronchoscopic debridement or surgical resection. Among this cohort, patients who received combined antifungal therapy and bronchoscopic debridement or surgical resection achieved a 12-month survival rate of 90.9% (with one death among the 11 patients who received combination therapy). However, the absence of a comparator group limits definitive conclusions regarding the independent contribution of each modality. Bronchoscopic treatment may enhance outcomes by removing purulent secretions, facilitating local drug administration, and alleviating bronchial stenosis or obstruction through procedures such as airway stent placement. Moreover, the combination of intravenous and local injection or nebulized AmB has shown favorable efficacy in some patients within this cohort. Additionally, 68.75% of patients with PM exhibited concomitant bronchial involvement, and two of the three deceased patients displayed extrapulmonary dissemination, suggesting that bilateral lung involvement or disseminated disease may contribute to a poor prognosis. This study has several limitations. Firstly, the small sample size (*n* = 16) precluded subgroup analyses and limited statistical power. Secondly, the retrospective, inpatient-only design introduced selection bias, potentially omitting mild or rapidly fatal cases. Thirdly, the decade-long enrollment period resulted in treatment heterogeneity, with evolving antifungals, bronchoscopic techniques, and supportive care complicating standardized efficacy comparisons. Fourthly, three cases were classified as probable rather than proven; despite excluding tuberculosis and aspergillosis, some diagnostic uncertainty remains. Lastly, the single-center design restricts generalizability.

## Conclusion

Pulmonary mucormycosis progresses rapidly and carries a high case-fatality rate. In this single-center, retrospective study of 16 patients from central China, the overall 12-month survival rate was 81.25%. Among those receiving a combined approach of systemic antifungal therapy and bronchoscopic intervention, the survival rate was 90.9% (10/11), although this observation requires validation in larger comparative studies. Diabetes mellitus was the dominant predisposing condition (81.25%), and bronchial involvement was frequent (68.75%). Hemoptysis (50%) may serve as a clinical diagnostic clue; radiologically, multiple small nodules, halo signs, and pleural involvement were common, whereas the classically described reversed halo sign was relatively infrequent. Diagnosis requires integration of clinical features, imaging, bronchoscopy, and histopathology; when tissue is unavailable, mNGS on clinical specimens may facilitate early confirmation. However, given the small sample size, the selection bias inherent to including only diagnosed inpatients, and the treatment heterogeneity over the decade, these findings must be interpreted with caution. Future multicenter prospective studies with larger cohorts are needed to validate our observations and establish standardized diagnostic and therapeutic algorithms.

## Data Availability

The raw data supporting the conclusions of this article will be made available by the authors, without undue reservation.
